# Acetyl Hexapeptide-8 in Cosmeceuticals—A Review of Skin Permeability and Efficacy

**DOI:** 10.3390/ijms26125722

**Published:** 2025-06-14

**Authors:** Julita Zdrada-Nowak, Agnieszka Surgiel-Gemza, Magdalena Szatkowska

**Affiliations:** Department of Cosmetology and Medical Biology, Wladyslaw Bieganski Collegium Medicum, Jan Dlugosz University, 42-200 Czestochowa, Poland; a.surgiel-gemza@ujd.edu.pl (A.S.-G.); m.szatkowska@ujd.edu.pl (M.S.)

**Keywords:** botox-like peptides, emulsions, skin barrier, skin permeation, acetyl hexapeptide-8

## Abstract

Biomimetic peptides represent a growing class of active ingredients in modern cosmeceuticals, designed to mimic the function of the naturally occurring peptides involved in skin homeostasis, repair, and regeneration. Among them, acetyl hexapeptide-8 (AH-8), often referred to as a “botox-like” peptide, has received considerable attention for its potential to dynamically reduce wrinkles through the modulation of neuromuscular activity. AH-8 is widely used in topical formulations intended for anti-aging effects, scar treatment, and skin rejuvenation. This review provides a comprehensive overview of the structure and proposed mechanisms of action of AH-8, with particular focus on its efficacy and skin penetration properties. Due to its hydrophilic nature and relatively large molecular size, AH-8 faces limited permeability through the lipophilic stratum corneum, making effective dermal delivery challenging. Formulation strategies such as oil-in-water (O/W) and multiple water-in-oil-in-water (W/O/W) emulsions have been explored to enhance its delivery, but the ability of AH-8 to reach neuromuscular junctions remains uncertain. Preclinical and clinical studies indicate that AH-8 may reduce wrinkle depth, improve skin elasticity, and enhance hydration. However, the precise biological mechanisms underlying these effects—particularly the peptide’s ability to inhibit muscle contraction when applied topically—remain incompletely understood. In some studies, AH-8 has also shown beneficial effects in scar remodeling and sebum regulation. Despite promising cosmetic outcomes, AH-8’s low skin penetration limits its bioavailability and therapeutic potential. This review emphasizes the need for further research on formulation science and delivery systems, which are essential for optimizing the effectiveness of peptide-based cosmeceuticals and validating their use as non-invasive alternatives to injectable treatments.

## 1. Introduction

Skin aging is driven by both extrinsic factors (e.g., UV radiation exposure) and intrinsic factors (e.g., telomere shortening and the Hayflick limit), which occur simultaneously. Undeniably, aging affects the cells of the all layers of the skin. Histological changes related to this process are observable in the dermis, including collagen, elastin, and hyaluronic acid degradation; the increased production of matrix metalloproteinases (MMPs); and decreased fibroblast protein synthesis. Mechanical stress in the extracellular matrix contributes to the disintegration of dermal cells [1]. These changes manifest clinically as depressions in the skin following the course of facial muscles, commonly referred to as wrinkles. Aging skin is dry, loose, and less elastic, with a compromised supportive function of the dermis, leading to visible telangiectasia [2,3]. Cosmetic and pharmaceutical companies are competing to develop new substances to mitigate the clinical symptoms of skin aging. However, not all substances exhibit comparable in vivo and in vitro efficacy. In some cases, their structure and skin penetration limitations prevent them from reaching their target sites. Peptides, which resemble physiological molecules and induce various effects in the skin, are particularly intriguing for researchers, although transdermal delivery remains challenging.

The complex architecture of the skin allows it to perform diverse functions, including thermoregulation, sensory perception, metabolism, and immune defense. However, its primary role is as a barrier limiting access to internal tissues. The skin forms a bidirectional barrier, preventing water and electrolyte loss while blocking harmful substances and pathogens. The first layer of skin interacting with the environment is the epidermis, composed of tightly packed cells that form a mechanical barrier restricting the penetration of substances, including the active ingredients in cosmetics and drugs, which is the main obstacle to delivering active ingredients to the skin in the form of externally applied products [4,5,6].

Biomimetic peptides have emerged as important active ingredients in modern cosmeceutical formulations, offering targeted approaches to skin rejuvenation, hydration, and wrinkle reduction. These synthetic compounds are designed to mimic naturally occurring peptides in the skin and modulate specific biological processes related to aging and repair. As interest in non-invasive alternatives to aesthetic procedures continues to grow, such peptides have become widely incorporated into topical skincare products.

Despite their increasing commercial use and consumer interest, the understanding remains limited of their actual skin permeability, bioavailability, and mechanisms of action, particularly when applied in over-the-counter formulations. Many of these peptides are hydrophilic and of relatively high molecular weight, making effective penetration through the lipophilic stratum corneum a major challenge. This raises questions about whether the observed effects are due to biological activity in deeper layers or result from surface-level interactions or formulation-related benefits.

This review addresses the need for a critical and evidence-based evaluation of the biomimetic peptides used in cosmetic dermatology. We focus on current data regarding skin penetration, delivery systems, and efficacy outcomes, aiming to clarify their potential and limitations. Understanding the delivery challenges and biological impact of these peptides is essential for improving product development and guiding future clinical and regulatory decisions.

In accordance with Regulation (EC) No. 1223/2009 of the European Parliament and of the Council, a cosmetic product is defined as “any substance or mixture intended to be placed in contact with the external parts of the human body (…) with a view exclusively or mainly to cleaning them, perfuming them, changing their appearance, protecting them, keeping them in good condition or correcting body odours.”

The term “cosmeceutical”, while commonly used in scientific and marketing contexts, does not have a legally recognized status in the European Union. In contrast, in the United States, the Food and Drug Administration (FDA) does not recognize “cosmeceuticals” as a distinct product category; products are regulated either as cosmetics or drugs, depending on their intended use. Nonetheless, in practice, the term is widely applied to cosmetic formulations that contain biologically active ingredients, such as peptides, and are intended to produce visible effects on the skin.

Therefore, in this review, the term “cosmeceutical” is used in the functional sense to describe topical cosmetic products that contain active peptides like acetyl hexapeptide-8 (AH-8) and demonstrate properties beyond basic skincare.

Three independent researchers conducted a literature search for articles published from December 2021 until December 2024 using three different databases (PubMed, Web of Science, and Google Scholar). A literature search was performed using the following keywords: argireline, acetyl hexapeptide-8, acetyl hexapeptide-3, skin permeation, botox-like peptides. Inclusion criteria were English language, any clinical study on the application acetyl hexapeptide-8 in vitro and in vivo including comparative clinical trials, randomized controlled trials, and reviews. Exclusion criteria were studies not associated with botox-like peptides treatment, skin permeation and mechanism of botox-like action, case reports, and letters to the editor.

## 2. Overview of Biomimetic Peptides in Cosmeceuticals

### 2.1. Biomimetic Peptides’ Properties

In recent years, interest in peptides as therapeutic agents has surged, especially in biomimetic peptides that mimic the action of physiological molecules such as parent proteins and peptides. Bioactive peptides are derived from plants, animals, microorganisms, and synthetic products. Furthermore, they can be rapidly and massively synthesized in laboratories. Their physiological functions are diverse, including hormonal activity, immune regulation, and antimicrobial, antiviral, and anticancer properties [Table 1]. Peptides have become a focal point in cosmetic research and applications due to their growing diversity, selectivity, efficacy, safety, and tolerability. The cosmetics industry continuously introduces new peptides, expanding their range of uses [7,8].

Biomimetic peptides play a significant role in combating aging, inflammation, cell proliferation and migration, angiogenesis, enzymatic regulation, pigmentation changes, and stimulating fibroblasts to synthesize extracellular matrix components, including collagen, elastin, and glycosaminoglycans [9].

Peptides’ diverse properties make them valuable in various medical fields, including dermatology, particularly in anti-aging applications. They are applied topically or intradermally to achieve localized effects. However, peptides are not inherently designed to penetrate the skin. Their high molecular weight and hydrophilicity pose significant barriers to transdermal delivery, prompting ongoing research into novel solutions [11].

A particularly intriguing group comprises neurotransmitter inhibitors synthesized as alternatives to botulinum toxin, aiming to avoid its side effects.

Botox, introduced in the 1990s for aesthetic use, was initially observed to smooth periorbital wrinkles during treatment for blepharospasm [12]. One of the primary causes of wrinkle formation is the stimulation of facial muscle fibers, which pull the skin inward, creating the characteristic appearance commonly known as expression lines. Consequently, an effective method to reduce the prominence of wrinkles involves directly lowering muscle activity or inhibiting the activity of neurons innervating these muscles, thereby decreasing their contractile response [13,14]. Currently, botulinum toxin type A is widely utilized to mitigate the signs of facial aging. However, its application is constrained by its high toxicity, with a lethal dose for humans [LD50] being ≈ 2500 mouse biological unit. This limitation underscores the need for the design and validation of non-toxic molecules, such as botox-like peptides. These molecules are designed to mimic the amino acid sequence of the synaptic protein SNAP-25, acting as specific neurosecretion inhibitors at micromolar concentrations [Table 2]. Additionally, they must demonstrate significant skin permeability, biocompatibility, and tolerability for safe and effective use in cosmetic and therapeutic applications [14].

Olsson et al. [15] analyzed the public interest in the substance acetyl hexapeptide-8. The study demonstrated an increasing interest in both the mentioned peptide and botulinum toxin. Acetyl hexapeptide-8 is gaining popularity as an alternative to botulinum neurotoxin, which may be attributed to its affordability, over-the-counter availability, and ease of application.

**Table 2:** Examples of neurotransmitter-inhibitor peptides (botox-like peptides) and their mechanisms of action.

Botox-like Peptide	Mechanism of Action
Argireline^®^ (Lipotec LTD, Barcelona, Spain), Acetyl Hexapeptide-8 (AH-8), or Acetyl Hexapeptide-3	A synthetic peptide developed as a topical mimic of botulinum toxin [16]. It inhibits neurotransmitter release by disrupting the formation and stabilization of the SNARE complex, which is essential for the docking of acetylcholine-releasing vesicles. Modeled after the N-terminus of the SNAP-25 protein, acetyl hexapeptide-8 competes with SNAP-25 for binding to vesicle-associated membrane protein (VAMP). This destabilizes the formation of the three-component soluble N-ethylmaleimide-sensitive factor attachment protein receptor (SNARE) complex, thereby inhibiting neuronal exocytosis. The lack of acetylcholine release during exocytosis prevents muscle contractions, which blocks the formation of expression wrinkles. Consequently, acetyl hexapeptide-8 inhibits acetylcholine release and reduces facial muscle contractions, effectively decreasing the appearance of expression lines [14,17,18].
Syn-Ake (Pentapharm, Basel, Switzerland) diacetyl tripeptide-3 or dipeptide diamino butyroyl benzylamide diacetate	A peptide fragment modeled after Waglerin-1, a protein derived from the venom of Wagler’s pit viper (*Tropidolaemus wagleri*). It mimics the action of Waglerin-1, which has been shown to block nicotinic acetylcholine receptors at the neuromuscular junction. By inhibiting these receptors, Syn-Ake effectively reduces muscle movements, such as facial expressions, which are responsible for dynamic wrinkles [9].
Leuphasyl (Lipotec S.A., Barcelona, Spain), Pentapeptide-18	Designed to modulate calcium channels by mimicking the action of enkephalins. It blocks the calcium channels in neurons in a similar manner to enkephalins, thereby inhibiting the release of acetylcholine. This mechanism disrupts neuromuscular transmission, which helps reduce muscle contractions and, consequently, minimizes the formation of expression lines and wrinkles [17].
Vialox^®^ (Cellular Skin, Rx, Sacramento, CA, USA), Pentapeptyd-3,	Designed to act similarly to tubocurarine, the primary active compound in curare. This peptide functions as a competitive antagonist at the postsynaptic acetylcholine receptor. By inhibiting the binding of acetylcholine to its receptor, Vialox prevents muscle contraction, which helps reduce the formation of wrinkles and fine lines [16].

### 2.2. Mechanism of Action of Botox-like Peptides, Using Acetylhexapeptide-8 as an Example

The application methods of botulinum toxin and biomimetic peptides differ, as botulinum toxin is administered intramuscularly, while botox-like biomimetic peptides are typically applied topically, occasionally intradermally, or even intramuscularly. The literature describes cases of Mycobacterium abscessus infection following the intradermal administration of acetylhexapeptide-8 [19] and botulinum toxin administration [20].

If significant amounts of acetylhexapeptide-8 penetrate the deeper layers of the skin and induce effects that modify the normal functions of the body without disease involvement, the anti-aging effect of this peptide may be considered therapeutic, and the substance would no longer qualify as a cosmetic product. According to The Federal Food, Drug, and Cosmetic Act (FD&C Act) [21], cosmetics are defined as “articles intended to be rubbed, poured, sprinkled, or sprayed on, introduced into, or otherwise applied to the human body… for cleansing, beautifying, promoting attractiveness, or altering the appearance” ([21], sec. 201(i)), while drugs are defined as “articles intended for use in the diagnosis, cure, mitigation, treatment, or prevention of disease” and “articles (other than food) intended to affect the structure or any function of the body of man or other animals” ([21], sec. 201(g)(1)).

Hwang et al. [22] designed muscle contraction tests in a co-culture system and the Caenorhabditis elegans model. The test, validated using botulinum toxin type A, demonstrated its dose-dependent inhibition of muscle cell contraction. When acetylhexapeptide-8 was tested, it was found that 100 ppm of AH-8 inhibited muscle contractions by 26%.

In connection with the above, questions arise regarding acetylhexapeptide-8:

- Do acetylhexapeptide-8 molecules have the ability to penetrate the skin and reach the muscles, their intended target?
- Does the concentration achieved in the muscle result in the inhibition of acetylcholine release?
- Will the substance, when applied to the skin, produce an anti-wrinkle effects through the inhibition of muscle contractions?
- Could the anti-wrinkle effects be induced through a different mechanism within the skin?

### 2.3. Permeation of Acetyl Hexapeptide-8—In Vitro Studies

To assess the ability of the peptide to permeate in vitro through the stratum corneum of human skin, an O/W emulsion containing 10% AH-8 was used. The peptide was placed in a donor chamber, and its content was measured in the receptor fluid after 2 h. The total peptide content in the receptor fluid amounted to a significant 30% of the amount applied to the membrane. This result indicates that acetyl hexapeptide-8 has the ability to permeate through the stratum corneum. In the same study, it was also demonstrated that 10% AH-8 in a face cream reduced the depth of wrinkles by 30% after 30 days in vivo [23].

In contrast to these findings, Kraeling et al. [24] studied the penetration of 10% acetyl hexapeptide-8 in an O/W emulsion through human skin in a diffusion chamber in vitro. After a 24 h exposure, the skin surface was washed to remove the unabsorbed peptide. The results of the skin penetration study showed that 0.22% of the applied peptide penetrated the stratum corneum, but the majority was removed from the skin (99.7%). No acetyl hexapeptide-8 was detected in the receptor solution, indicating that the substance did not penetrate through the skin.

The permeability of AH-8 through the skin in vitro from a multiple W/O/W emulsion was compared with simple O/W and W/O emulsions. Comparison of the cumulative doses of the substance clearly showed that AH-8 permeated more quickly and to a significantly greater extent from the multiple W/O/W emulsion and the O/W emulsion, whereas it was undetectable from the W/O emulsion. However, a difference was observed between the multiple W/O/W emulsion and the O/W emulsion in the cumulative permeated dose of the peptide after 8 h. This suggested that the multiple emulsion significantly enhanced the skin penetration of acetyl hexapeptide-8 [25].

Kraeling et al. [24] and Hoppel et al. [25] performed tape stripping of skin removed from a diffusion chamber to determine the concentration gradient of the AH-8 in the epidermis. The amount of acetyl hexapeptide-8 in the stratum corneum decreased as it approached the living layers of the epidermis. The highest concentration of the substance was found in the outer layers of the stratum corneum.

The delivery of hydrophilic macromolecules such as peptides through the skin is difficult due to the lipophilic nature of the stratum corneum, and, therefore, the permeation of AH-8 through all layers of the skin may be impossible [26]. An in vitro study of the release of acetyl hexapeptide-8 from an O/W emulsion showed absorption below 50%, which is satisfactory for a topically applied cosmetic product [27].

The suitability of multiple W/O/W emulsions as skin delivery systems for AH-8 was studied. Human skin and receptor solutions with acidic (pH 2.7) and alkaline (pH 7.4) conditions were used as membranes in a Franz diffusion chamber. After 8 h, AH-8 was detected in the alkaline receptor solution to a small extent (2–3%), while its permeability through the skin in the acidic environment was significantly higher. This indicates that the solubility of the peptide is much better in an acidic environment [28]. This is crucial for the formulation of cosmetic products and maintaining the product’s pH at the physiological skin level, allowing more of the substance to permeate the epidermis.

In a study by Zhang et al. [29], the in vitro skin permeation of hydrophilic peptides of various molecular weights was analyzed with the use of porcine ear skin. For acetyl hexapeptide-3 the authors demonstrated that the cumulative amount of acetyl hexapeptide-3 permeated over 24 h was over 31-fold higher after microneedle treatment compared to the passive flux across untreated skin (0.44 ± 0.12 μmoL/cm·h vs. 0.014 ± 0.002 μmoL/cm·h, respectively). The study shows that microneedles provide an attractive route for the delivery of low-molecular-weight peptides to the skin.

## 3. Effect of Acetyl Hexapeptide-8 on the Skin

### 3.1. Skin as a Delivery Barrier

The transdermal delivery of active substances is currently gaining significant attention in clinical medicine and the cosmetic industry, as it can significantly improve the transdermal penetration of hydrophilic compounds. Delivering active substances through the epidermal barrier is challenging due to its structure. Some substances undergo degradation on the surface of the stratum corneum, while others break down during transdermal penetration. As a result, only a small percentage of these substances can reach deeper layers. This may lead to the low bioavailability of active substances and, consequently, unsatisfactory therapeutic outcomes. The mechanical puncturing of the skin with fine needles disrupts the continuity of the epidermis, allowing for increased penetration. Studies on transdermal drug delivery to various skin layers using microneedling have confirmed increased concentrations of lipophilic and hydrophilic pharmaceuticals as well as macromolecules in the layers below the stratum corneum [30,31]. Additionally, the preparation can be injected, resulting in nearly 100% intradermal delivery, where the substance is able to act effectively.

Various factors influence the ability of substances to penetrate the skin: the physicochemical properties of the substance; the type of emulsion; the integrity, thickness, and composition of the skin; skin metabolism; the site, area, and duration of application; transdermal delivery properties; and the formation of a local reservoir at the site of application [32]. It appears that acetyl hexapeptide-8 penetrates the layers of the epidermis, but there is a low chance of it penetrating the dermis. Therefore, the transdermal delivery of AH-8 to induce a paralyzing effect in muscles is likely impossible.

### 3.2. Effectiveness of Acetyl Hexapeptide-8 in Wrinkle Reduction

There are reports on the anti-wrinkle effectiveness of AH-8. Wang et al. [33] conducted a study evaluating the efficacy of a formulation containing 10% acetyl hexapeptide-8. After 4 weeks of daily application of the product to the facial skin, there was a 49% reduction in wrinkle depth. In vivo studies on mice also showed improvements in skin tissue morphology and collagen fiber proliferation.

In mice that were given subcutaneous injections of hexapeptide-8 daily for 6 weeks, an increase in type I collagen fibers and a decrease in type III collagen fibers were observed. According to the authors of the study, the improvement in these parameters was attributed to the reduction in muscle activity around wrinkles and the stimulation of collagen fiber growth [34].

In vivo application studies of a 10% AH-8 cream to the skin showed that skin elasticity parameters, measured by cutometry, did not significantly change after 4 weeks of daily application of the placebo or the 10% AH-8 formulation. Additionally, the peptide cream did not statistically significantly increase the water content in the stratum corneum compared to the placebo [35]. Raikou et al. [36] demonstrated that a cream containing 10% AH-8 slightly reduced skin roughness and decreased TEWL (transepidermal water loss) after 20 days of use. These studies assessed the biomechanical parameters of the epidermis, not the muscle contraction potential in vivo.

While AH-8 formulations improve the appearance of the skin and reduce the depth of wrinkles, none of the aforementioned in vivo application studies confirmed the peptide’s inhibitory effect on muscle contractions and its role in smoothing wrinkles. It can be hypothesized that a different mechanism, related to the peptide’s permeation into the epidermis, may be responsible for these effects.

### 3.3. Effect of Acetyl Hexapeptide-8 on the Appearance of Scars

Scarring occurs as a result of the wound healing process. The causes of wounds include mechanical, chemical, and thermal injuries, as well as inflammatory skin conditions, such as acne vulgaris and other dermatoses. Wound healing is a multi-stage process consisting of several distinct phases: hemostatic, inflammatory, proliferative (growth), and remodeling (maturation). Under normal healing conditions, normative scars form; however, when the process is disrupted, such as through prolonged inflammation, atrophic or hypertrophic scars, including keloids and hypertrophic scars, may develop [37].

In a study by Olsson et al. [38], the impact of scars on adolescents and adults after cancer treatment was examined. Using a questionnaire addressing various aspects of mental health and self-acceptance, the authors observed that the risk of feeling less attractive due to scars was higher among cancer survivors, regardless of gender, compared to the control group.

Scar reduction is achieved through various methods, including natural remedies, pharmacological therapies, surgery, laser treatments, microneedling, and fractional radiofrequency microneedling, both as monotherapy and in combination therapies [39,40,41,42,43].

Promising results in the treatment of immature scars have been reported with botulinum toxin type A (BoNTA). This neurotoxin reduces muscle tension at the wound edges, has anti-inflammatory effects, and supports angiogenesis. Due to its multifaceted action, BoNTA is used both for the prevention and treatment of excessive scarring [44].

Considering the above, a study was conducted to assess the therapeutic efficacy of a topical gel formulation containing acetyl hexapeptide-8. The aim of the study was to improve the appearance of scars and skin imperfections. The study involved 26 patients with scars in various locations, including the right and left sides of the face, forehead, and chin. Parameters measured included skin hydration, elasticity, and sebum levels at the scar site compared to the surrounding undamaged skin. A slight decrease in hydration levels was observed, along with a significant increase in skin elasticity in the treated area and a substantial reduction in sebum production at the scar site relative to the surrounding area. Photographs taken before and after the therapy showed significant improvement in scars resulting from surgical interventions related to cancer treatment, cyst removal, or mole excisions. No worsening of the treated areas or significant adverse effects were noted. Additional benefits included reductions in wrinkles, puffiness, and dark circles under the eyes, as well as a reduction in the size of enlarged sebaceous gland openings by up to 85% [45].

### 3.4. Application of Acetyl Hexapeptide-8 in Acne Vulgaris Therapy

Acne vulgaris is a chronic, multifactorial inflammatory disease of the pilosebaceous unit that affects a significant portion of the adolescent population, as well as adults [30]. A key element in its pathogenesis is the excessive activity of the sebaceous glands and sebum production, influenced by various factors including androgens, neuropeptide signaling, and local inflammatory mediators [31,32].

In recent years, there has been growing interest in the modulation of sebaceous gland function as a potential therapeutic target [33,34]. In this context, particular attention has been given to botulinum toxin type A (BoNT-A) and acetyl hexapeptide-8—a biomimetic peptide often referred to as “botox-like”. Both compounds have the ability to modulate the activity of cutaneous nerve endings, making them potentially effective in the treatment of seborrhea and acne [35,36].

BoNT-A exerts its action by inhibiting the release of acetylcholine at the neuroglandular and neuromuscular synapses. In the skin, this results in reduced cholinergic stimulation of sebaceous glands, thereby decreasing sebum production. AH-8, in turn, blocks the SNARE complex in a manner analogous to BoNT-A; however, its effect is limited to the surface of the skin—when applied topically, it may reduce muscle tension and, as suggested by some studies, indirectly influence sebaceous gland activity [37].

The use of botulinum toxin type A (BoNT-A) in the treatment of acne is an innovative approach gaining traction in aesthetic medicine and clinical dermatology. Traditionally employed in the management of dynamic wrinkles, dystonia, and chronic migraines, BoNT-A has also demonstrated the ability to reduce sebum production by modulating the cholinergic innervation of sebaceous glands [37].

BoNT-A acts by blocking the release of acetylcholine from nerve endings through the hydrolysis of SNAP-25, a key component of the SNARE complex responsible for neurotransmitter exocytosis. Acetylcholine plays a crucial role in stimulating sebaceous gland activity—its inhibition leads to a decrease in sebum production and a reduction in seborrhea, which is one of the factors contributing to the exacerbation of acne lesions [37].

In a study conducted by Shah et al. [38], the subcutaneous administration of BoNT-A in the forehead region of patients with seborrhea showed a significant reduction in sebum production, lasting up to 3 months post-injection. Similar results were reported by Li et al. [39], who observed that both human sebaceous glands in vivo and sebocytes in vitro expressed the nicotinic acetylcholine receptor α7 (nAchRα7). Additionally, acetylcholine increased lipid synthesis in a dose-dependent manner. Incubation of sebocytes with α-bungarotoxin, a competitive nAchR antagonist, prevented acetylcholine from inducing lipid synthesis. In a double-blind, placebo-controlled study involving 20 healthy volunteers, a significant reduction in sebum production was observed on the botulinum-toxin-treated side in volunteers with oily skin.

Although a standardized protocol for acne treatment using botulinum toxin type A (BoNT-A) has not yet been universally established, the available literature commonly describes a treatment regimen involving the administration of 10 to 30 units of BoNT-A per affected area, typically diluted in 1–2 mL of 0.9% sodium chloride solution. Injections are usually performed in regions with the highest sebaceous activity, such as the forehead, chin, nose, and cheeks. The recommended technique consists of superficial intradermal or very shallow subcutaneous injections spaced approximately 1 cm apart. The treatment is generally repeated every 3 to 4 months, depending on the patient’s individual response and the recurrence of seborrhea [38,40].

Due to its structural and functional similarity to botulinum toxin, AH-8 is considered to have sebosuppressive potential based on its mechanism of action. It inhibits the release of acetylcholine at nerve endings, a neurotransmitter that plays a key role in regulating sebaceous gland function and cutaneous inflammation. By reducing acetylcholine availability, AH-8 may help limit sebum production, erythema, and the formation of acne lesions. Although most studies on AH-8 have focused on its anti-wrinkle properties, emerging evidence suggests its beneficial effects on acne-prone skin [15].

Although AH-8 is often referred to as a “botox-like” peptide due to its proposed mechanism of inhibiting muscle contraction, it is important to distinguish between its epidermal-level effects and the intended neuromuscular action. The stratum corneum presents a significant barrier to the transdermal delivery of large, hydrophilic molecules such as AH-8. As such, most formulations are likely to exert their effects at the superficial skin level, including improvements in hydration and skin elasticity as well as reductions in superficial wrinkles, rather than reaching the depth required to modulate neuromuscular activity. Evidence for the inhibition of synaptic transmission in facial muscles via topical application remains limited and largely hypothetical. Therefore, while AH-8 shows promising cosmetic benefits, its mechanism of action is likely multifactorial and predominantly localized to the epidermis and upper dermis, unless delivered through alternative methods such as injection.

## 4. Conclusions

Due to the lipophilic properties of the stratum corneum, achieving therapeutic concentrations of hydrophilic active substances such as acetyl hexapeptide-8, which poorly penetrate the skin, is challenging. The lipophilicity of the molecule that is to be applied to the skin plays a crucial role in penetration. Given the specific structure of the epidermis, not all molecules can pass through, but penetration can be facilitated by auxiliary substances and the chemical nature of the emulsion. The penetration of AH-8 is enhanced by O/W emulsions and multiple W/O/W emulsions. However, these are not sufficiently effective to achieve penetration into the deeper layers of the dermis, which is necessary to inhibit muscle contraction. The simplest way of achieving such effects would be via injection. In vivo studies indicate the effective anti-wrinkle action of acetyl hexapeptide-8. The efficacy of emulsions containing acetyl hexapeptide-8 has been studied by numerous research centers, but reports on penetration are limited, and even fewer have addressed the actual mechanism of action in the skin. Further research is needed to clarify the anti-wrinkle mechanism of acetyl hexapeptide-8. There are still many unanswered questions that may lead researchers toward a better understanding of the action of botox-like peptides, ensuring they are both safe and effective. This review highlights the multifunctional properties of acetyl hexapeptide-8, suggesting the potential to expand its indications for safe use, serving as an alternative to botulinum toxin.

## Figures and Tables

**Table 1:** Classification of peptides based on their mechanism of action and examples of substances [9,10].

Group of Peptides	Mechanism of Action	Substances
Signal peptides	Bind to cell receptors to stimulate fibroblast division	Palmitoyl oligopeptide, palmitoyl pentapeptide-3
Enzyme-inhibiting peptides	Inhibit enzymatic activity, e.g., MMPs	Dipeptide-2, tripeptide-2
Transport peptides	Transport substances essential for skin metabolic processes	Copper tripeptide-1
Neurotransmitter inhibitors	Inhibit neurotransmitter transmission at synapses, relaxing muscles	Acetyl hexapeptide-8, pentapeptide-18

## Data Availability

No new data were created or analyzed in this study. Data sharing is not applicable to this article.

## References

[1] Kohl E., Steinbauer J., Landthaler M., Szeimies R.M. (2011). Skin Ageing. J. Eur. Acad. Dermatol. Venereol..

[2] Ramos-e-Silva M., Celem L.R., Ramos-e-Silva S., Fucci-da-Costa A.P. (2013). Anti-Aging Cosmetics: Facts and Controversies. Clin. Dermatol..

[3] Lee J.Y., Kim Y.K., Seo J.Y., Choi C.W., Hwang J.S., Lee B.G., Chang I.S., Chung J.H. (2008). Loss of Elastic Fibers Causes Skin Wrinkles in Sun-Damaged Human Skin. J. Dermatol. Sci..

[4] Gorzelanny C., Mess C., Schneider S.W., Huck V., Brandner J.M. (2020). Skin Barriers in Dermal Drug Delivery: Which Barriers Have to Be Overcome and How Can We Measure Them?. Pharmaceutics.

[5] Tsakovska I., Pajeva I., Al Sharif M., Alov P., Fioravanzo E., Kovarich S., Worth A.P., Richarz A.-N., Yang C., Mostrag-Szlichtyng A. (2017). Quantitative Structure-Skin Permeability Relationships. Toxicology.

[6] Zhang L., Dong Z., Liu W., Wu X., He H., Lu Y., Wu W., Qi J. (2022). Novel Pharmaceutical Strategies for Enhancing Skin Penetration of Biomacromolecules. Pharmaceuticals.

[7] Kang L., Han T., Cong H., Yu B., Shen Y. (2022). Recent Research Progress of Biologically Active Peptides. BioFactors.

[8] Mine Y., Munir H., Nakanishi Y., Sugiyama D. (2016). Biomimetic Peptides for the Treatment of Cancer. Anticancer Res..

[9] Zhang L., Falla T.J. (2009). Cosmeceuticals and Peptides. Clin. Dermatol..

[10] Pai V.V., Bhandari P., Shukla P. (2017). Topical Peptides as Cosmeceuticals. Indian J. Dermatol. Venereol. Leprol..

[11] Sommer E., Neubert R.H.H., Mentel M., Tuchscherer B., Mrestani Y., Wohlrab J. (2018). Dermal Peptide Delivery Using Enhancer Molecules and Colloidal Carrier Systems. Part III: Tetrapeptide GEKG. Eur. J. Pharm. Sci. Off. J. Eur. Fed. Pharm. Sci..

[12] Klein A.W. (2001). Botulinum Toxins. Introduction. Semin. Cutan. Med. Surg..

[13] Wongrattanakamon P., Nimmanpipug P., Sirithunyalug B., Jiranusornkul S. (2018). Molecular Modeling Elucidates the Cellular Mechanism of Synaptotagmin-SNARE Inhibition: A Novel Plausible Route to Anti-Wrinkle Activity of Botox-like Cosmetic Active Molecules. Mol. Cell. Biochem..

[14] Fields K., Falla T.J., Rodan K., Bush L. (2009). Bioactive Peptides: Signaling the Future. J. Cosmet. Dermatol..

[15] Olsson S.E., Sreepad B., Lee T., Fasih M., Fijany A. (2024). Public Interest in Acetyl Hexapeptide-8: Longitudinal Analysis. JMIR Dermatol..

[16] Lupo M.P., Cole A.L. (2007). Cosmeceutical Peptides. Dermatol. Ther..

[17] Husein El Hadmed H., Castillo R.F. (2016). Cosmeceuticals: Peptides, Proteins, and Growth Factors. J. Cosmet. Dermatol..

[18] Fu F.N., Lomneth R.B., Cai S., Singh B.R. (1998). Role of Zinc in the Structure and Toxic Activity of Botulinum Neurotoxin. Biochemistry.

[19] Chen C.-F., Liu J., Wang S.-S., Yao Y.-F., Yu B., Hu X.-P. (2021). Mycobacterium Abscessus Infection after Facial Injection of Argireline: A Case Report. World J. Clin. Cases.

[20] Fang R., Sun Q. (2020). Mycobacterium Abscessus Infections Following Injection of Botulinum Toxin. J. Cosmet. Dermatol..

[21] United States Food and Drug Administration (FDA) (1938). Federal Food, Drug, and Cosmetic Act (FD&C Act), Section 201. https://www.fda.gov/regulatory-information/laws-enforced-fda/federal-food-drug-and-cosmetic-act-fdc-act.

[22] Hwang W., Kim D., Kwon O.S., Kim Y.-S., Ahn B., Kang N.-G. (2020). Topical Application of Zanthoxylum Piperitum Extract Improves Lateral Canthal Rhytides by Inhibiting Muscle Contractions. Sci. Rep..

[23] Blanes-Mira C., Clemente J., Jodas G., Gil A., Fernández-Ballester G., Ponsati B., Gutierrez L., Pérez-Payá E., Ferrer-Montiel A. (2002). A Synthetic Hexapeptide (Argireline) with Antiwrinkle Activity. Int. J. Cosmet. Sci..

[24] Kraeling M.E.K., Zhou W., Wang P., Ogunsola O.A. (2015). In Vitro Skin Penetration of Acetyl Hexapeptide-8 from a Cosmetic Formulation. Cutan. Ocul. Toxicol..

[25] Hoppel M., Reznicek G., Kählig H., Kotisch H., Resch G.P., Valenta C. (2015). Topical Delivery of Acetyl Hexapeptide-8 from Different Emulsions: Influence of Emulsion Composition and Internal Structure. Eur. J. Pharm. Sci. Off. J. Eur. Fed. Pharm. Sci..

[26] Kalluri H., Banga A.K. (2011). Transdermal Delivery of Proteins. AAPS PharmSciTech.

[27] Ruiz M.A., Clares B., Morales M.E., Cazalla S., Gallardo V. (2007). Preparation and Stability of Cosmetic Formulations with an Anti-Aging Peptide. J. Cosmet. Sci..

[28] Hoppel M., Juric S., Reznicek G., Wirth M., Valenta C. (2015). Multiple W/O/W Emulsions as Dermal Peptide Delivery Systems. J. Drug Deliv. Sci. Technol..

[29] Zhang S., Qiu Y., Gao Y. (2014). Enhanced Delivery of Hydrophilic Peptides in Vitro by Transdermal Microneedle Pretreatment. Acta Pharm. Sin. B.

[30] Zduńska K., Kołodziejczak A., Rotsztejn H. (2018). Is Skin Microneedling a Good Alternative Method of Various Skin Defects Removal. Dermatol. Ther..

[31] Zasada M., Markiewicz A., Drożdż Z., Mosińska P., Erkiert-Polguj A., Budzisz E. (2019). Preliminary Randomized Controlled Trial of Antiaging Effects of L-Ascorbic Acid Applied in Combination with No-Needle and Microneedle Mesotherapy. J. Cosmet. Dermatol..

[32] Gorouhi F., Maibach H.I. (2009). Role of Topical Peptides in Preventing or Treating Aged Skin. Int. J. Cosmet. Sci..

[33] Wang Y., Wang M., Xiao X.S., Pan P., Li P., Huo J. (2013). The Anti Wrinkle Efficacy of Synthetic Hexapeptide (Argireline) in Chinese Subjects. J. Cosmet. Laser Ther. Off. Publ. Eur. Soc. Laser Dermatol..

[34] Wang Y., Wang M., Xiao X.S., Huo J., Zhang W.D. (2013). The Anti-Wrinkle Efficacy of Argireline. J. Cosmet. Laser Ther. Off. Publ. Eur. Soc. Laser Dermatol..

[35] Tadini K.A., Mercurio D.G., Campos P.M.B.G.M. (2015). Acetyl Hexapeptide-3 in a Cosmetic Formulation Acts on Skin Mechanical Properties—Clinical Study. Braz. J. Pharm. Sci..

[36] Raikou V., Varvaresou A., Panderi I., Papageorgiou E. (2017). The Efficacy Study of the Combination of Tripeptide-10-Citrulline and Acetyl Hexapeptide-3. A Prospective, Randomized Controlled Study. J. Cosmet. Dermatol..

[37] Fernández-Guarino M., Bacci S., Pérez González L.A., Bermejo-Martínez M., Cecilia-Matilla A., Hernández-Bule M.L. (2023). The Role of Physical Therapies in Wound Healing and Assisted Scarring. Int. J. Mol. Sci..

[38] Olsson M., Enskär K., Steineck G., Wilderäng U., Jarfelt M. (2018). Self-Perceived Physical Attractiveness in Relation to Scars Among Adolescent and Young Adult Cancer Survivors: A Population-Based Study. J. Adolesc. Young Adult Oncol..

[39] Ghazawi F.M., Zargham R., Gilardino M.S., Sasseville D., Jafarian F. (2018). Insights into the Pathophysiology of Hypertrophic Scars and Keloids: How Do They Differ?. Adv. Ski. Wound Care.

[40] Fang Q.-Q., Chen C.-Y., Zhang M.-X., Huang C.-L., Wang X.-W., Xu J.-H., Wu L.-H., Zhang L.-Y., Tan W.-Q. (2017). The Effectiveness of Topical Anti-Scarring Agents and a Novel Combined Process on Cutaneous Scar Management. Curr. Pharm. Des..

[41] Eilers R.E.J., Ross E.V., Cohen J.L., Ortiz A.E. (2016). A Combination Approach to Surgical Scars. Dermatol. Surg. Off. Publ. Am. Soc. Dermatol. Surg..

[42] Gauglitz G.G., Korting H.C., Pavicic T., Ruzicka T., Jeschke M.G. (2011). Hypertrophic Scarring and Keloids: Pathomechanisms and Current and Emerging Treatment Strategies. Mol. Med..

[43] Mustoe T.A., Cooter R.D., Gold M.H., Hobbs F.D.R., Ramelet A.-A., Shakespeare P.G., Stella M., Téot L., Wood F.M., Ziegler U.E. (2002). International Clinical Recommendations on Scar Management. Plast. Reconstr. Surg..

[44] Chambers A., Téot L., Mustoe T.A., Middelkoop E., Gauglitz G.G. (2020). Treatment of Immature Scars with Botulinum Toxin. Textbook on Scar Management: State of the Art Management and Emerging Technologies.

[45] Palmieri B., Noviello A., Corazzari V., Garelli A., Vadala M. (2020). Skin Scars and Wrinkles Temporary Camouflage in Dermatology and Oncoesthetics: Focus on Acetyl Hexapeptide-8. La Clin. Ter..

